# Saturation transfer MRI is sensitive to neurochemical changes in the rat brain due to chronic unpredictable mild stress

**DOI:** 10.1038/s41598-021-97991-0

**Published:** 2021-09-24

**Authors:** Anna Pankowska, Agata Chudzik, Tymoteusz Słowik, Artur Łazorczyk, Katarzyna Kochalska, Marta Andres-Mach, Wilfred W. Lam, Radosław Pietura, Radosław Rola, Greg J. Stanisz, Anna Orzyłowska

**Affiliations:** 1 Department of Radiography, Medical University of Lublin, Lublin, Poland; 2 Department of Neurosurgery and Paediatric Neurosurgery, Medical University of Lublin, Jaczewskiego 8, 20-090 Lublin, Poland; 3 Experimental Medicine Center, Medical University of Lublin, Lublin, Poland; 4 Isobolographic Analysis Laboratory, Institute of Rural Health, Lublin, Poland; 5 Physical Sciences, Sunnybrook Research Institute, Toronto, ON Canada; 6 Department of Medical Biophysics, University of Toronto, Toronto, ON Canada

**Keywords:** Depression, Diseases of the nervous system, Stress and resilience, Magnetic resonance imaging

## Abstract

Chemical exchange saturation transfer (CEST) MRI was performed for the evaluation of cerebral metabolic changes in a rat model of depressive-like disease induced by chronic unpredictable mild stress (CUMS). CEST Z-spectra were acquired on a 7 T MRI with two saturation B_1_ amplitudes (0.5 and 0.75 µT) to measure the magnetization transfer ratio (MTR), CEST and relayed nuclear Overhauser effect (rNOE). Cerebral cortex and hippocampus were examined in two groups of animals: healthy control (n = 10) and stressed (n = 14), the latter of which was exposed to eight weeks of the CUMS protocol. The stressed group Z-spectrum parameters, primarily MTRs, were significantly lower than in controls, at all selected frequency offsets (3.5, 3.0, 2.0, − 3.2, − 3.6 ppm) in the cortex (the largest difference of ~ 3.5% at − 3.6 ppm, p = 0.0005) and the hippocampus (MTRs measured with a B_1_ = 0.5 µT). The hippocampal rNOE contributions decreased significantly in the stressed brains. Glutamate concentration (assessed using ELISA) and MTR at 3 ppm correlated positively in both brain regions. GABA concentration also correlated positively with CEST contributions in both cerebral areas, while such correlation with MTR was positive in hippocampus, and nonsignificant in cortex. Results indicate that CEST is sensitive to neurometabolic changes following chronic stress exposure.

## Introduction

Over the last two decades, many attempts have been made to use magnetic resonance imaging (MRI) to evaluate structural^1,2^, functional^3^ and metabolic changes^4,5^ in the brain affected by mood disorders. Previous studies have demonstrated reduced hippocampal volume in patients diagnosed with depression by about 20% compared to healthy controls^6,7^. Moreover, other brain structures such as the amygdala and prefrontal cortex can also be vulnerable to damage from chronic stress exposure^8^.

Functional MRI (fMRI) studies in depressive patients report pathological patterns of functional network organization^9^. A resting-state fMRI study performed by Meng et al*.*^10^ have shown reduced global network efficiency and an increased characteristic path length in the depressed group, while other research^11^ has found functional connectivity strength to be lower in the bilateral ventral medial prefrontal cortex and ventral anterior cingulate cortex regions of depressed brains than in corresponding areas in a healthy group.

Magnetic resonance spectroscopy (MRS) studies performed in depressive patients show decreased metabolic ratios (NAA/Cr, Cho/Cr and Ins/Cr) in the prefrontal region^12^ compared to healthy subjects. Moreover, in many animal experiments that mimic major depressive disorders^13–15^, MRS reveals an abnormal decrease in glutamine (Gln), glutamate (Glu), γ-aminobutyric acid (GABA) and *N*-acetylaspartate (NAA) levels in hippocampal and cortical areas. MRS has become the most frequently used method in metabolic studies, mostly due to its high specificity^16,17^. However, low MRS signal originating from brain metabolites severely limits its resolution, while B_0_ field inhomogeneities make it less accurate in deep brain structures^18^. Therefore, it is often difficult to assess changes in brain metabolism in structures other than the cortex.

In recent years, there has been a growing interest in using chemical exchange saturation transfer (CEST) imaging to probe brain metabolites^19,20^, as it offers improved resolution compared to MRS, although at the cost of specificity. CEST measurements are based on contrast from the transfer of magnetization from saturated labile hydrogen nuclei in molecules of various compounds to those in water molecules through chemical exchange and often acquired as spectra (denoted Z-spectra) as a function of saturation frequency offset^21,22^. Assessing the process of protons exchange between water and such CEST-sensitive compounds as neurotransmitters can become an important factor in predicting later changes in the brain that are responsible for depressive disorders. Amide, guanidinium and hydroxyl groups are detectable using CEST imaging^23–26^ and, more importantly, even subtle changes in the concentrations of compounds containing these groups are detectable^27^. Moreover, the exchange of magnetization between aliphatic groups, such as methine, and water can also be detected upfield of the water resonance^28,29^.

CEST as an endogenous contrast has been used in various studies, both in animal models of disease and clinical trials. Evaluation of tumour malignancy^30,31^, differentiation between tumour progression and necrosis^32,33^, the examination of muscle physiology^34,35^ and glutamate imaging in epilepsy^36^ are just some successful examples. As most of the brain metabolites affected by mood disorders, such as anxiety and depression, display the CEST effect^37^, it would be worthwhile to use CEST to investigate neurochemical processes in the depressive brain.

In this study, we evaluated characteristics of the saturation transfer data such as the magnetization transfer ratio (MTR), which is a non-specific measure of T_1_/T_2_ ratio, magnetization transfer (MT) and CEST effects, but also CEST and relayed nuclear Overhauser effect (rNOE) contributions to the Z-spectra to investigate brain metabolic changes related to depressive disorder with the use of the chronic unpredictable mild stress (CUMS) rat model. We acquired the saturation transfer MRI, T_1_ maps and MRS data from two groups of animals: stressed and control, from two selected brain regions: hippocampus and cortex, and compared them to biochemically assessed GABA and glutamate levels. We hypothesized that CEST is sensitive to neurochemical changes following chronic stress exposure.

## Results

**Figure 1:**
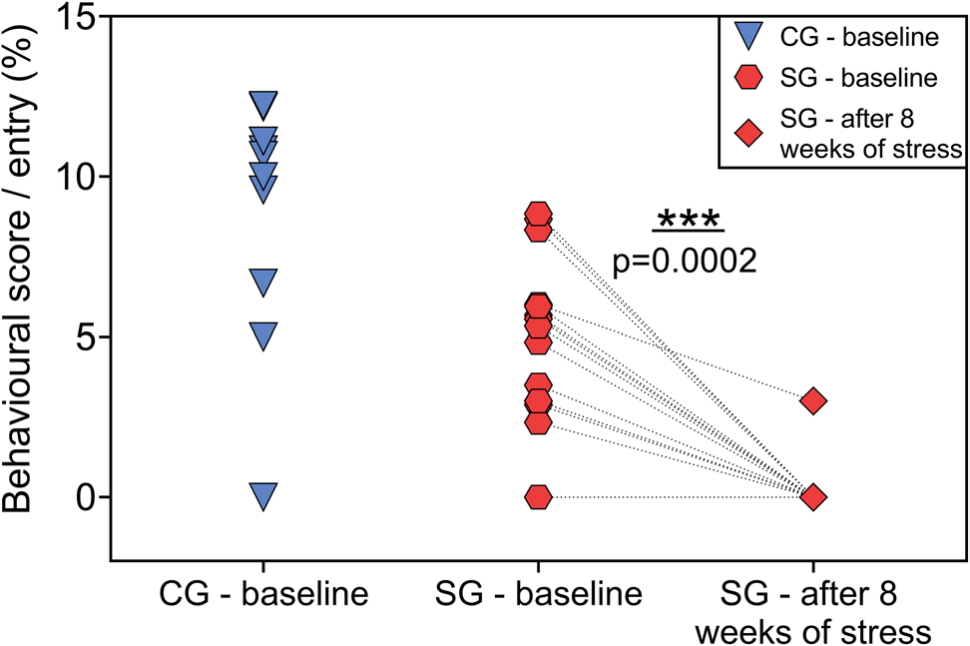
Elevated plus maze (EPM) behavioural test results. First two columns show baseline scores per entry into the open arms of the EPM of each animal from the control (CG) and stressed (SG) group. Results presented in last column are scores gained by animals from stressed group after eight weeks of stress. The dotted lines indicate the change between before and after CUMS protocol for individual animal from SG group. P-value (p = 0.0002) was obtained using a non-parametric Wilcoxon test for paired samples.

In the elevated plus maze behavioural test, the control group of animals showed an exploration scores per entry of 8.8 ± 1.2% (mean ± SEM), and the group of animals to be stressed at baseline showed the behavioural score/entry of 5.1 ± 0.7%. After eight weeks of CUMS, 13 of 14 animals in the latter group completely avoided exploration and stayed in the dark arms of the maze. A non-parametric Wilcoxon test for paired samples showed significant changes in exploration scores/entry for the stressed group before and after CUMS (p = 0.0002). Results are presented in Fig. 1.

**Figure 2:**
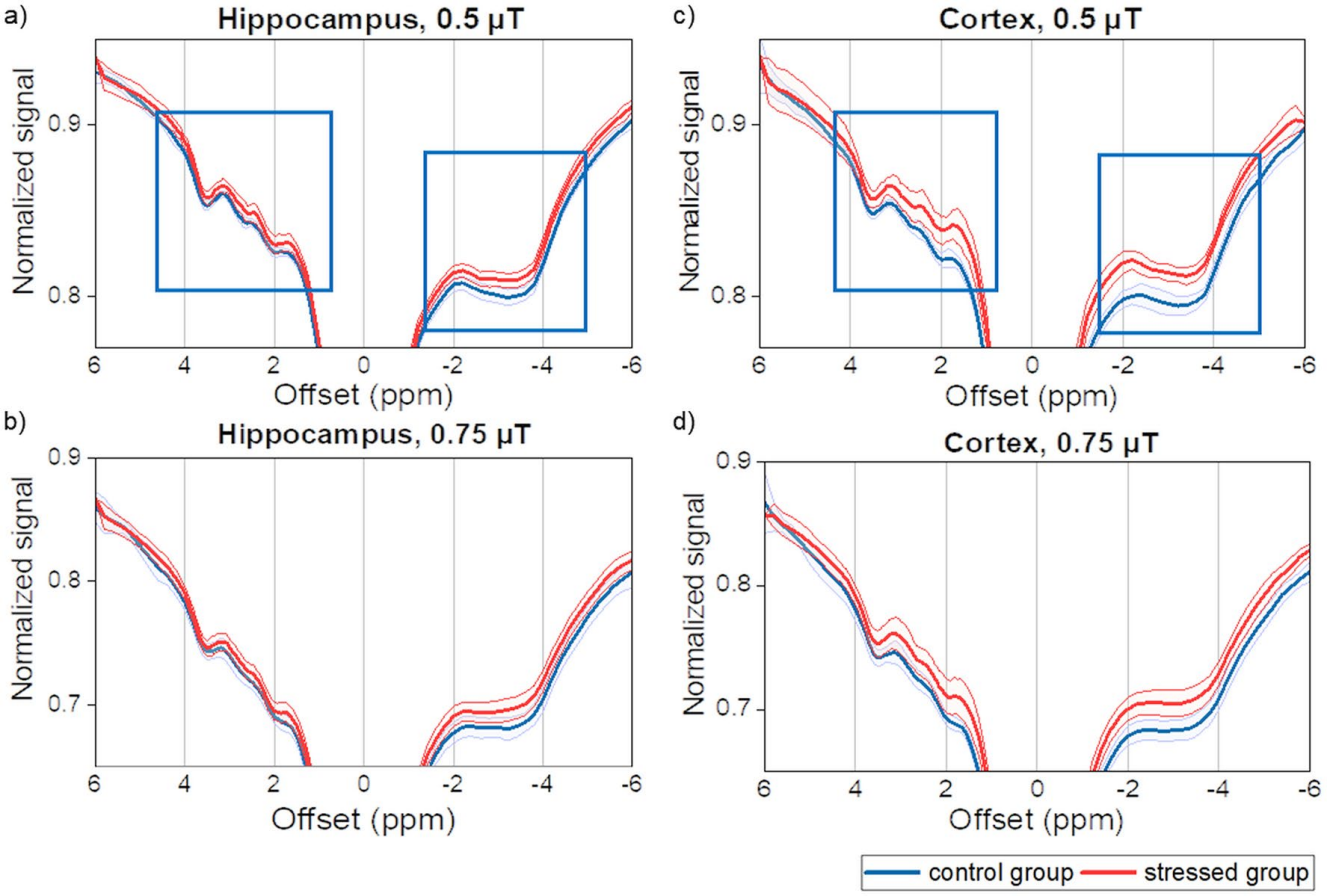
Mean Z-spectra from the (**a**,**b**) hippocampus and (**c**,**d**) cortex acquired with saturation B_1_s of 0.5 and 0.75 µT show a lowering of CEST contrast (an increase in signal) after stress, mainly in the cortex. The ranges with the largest differences are marked with blue rectangles. The solid lines are the average Z-spectra for all the animals in the group, whereas the shaded areas illustrate one standard deviation.

Figure 2 shows the average CEST Z-spectra for both the hippocampus and cortex at different saturation amplitudes, B_1_, (0.5 and 0.75 µT) and clearly demonstrates lower CEST contrast in the stressed group compared to the control. It is most pronounced in the ranges where the SD of the mean spectra do not overlap: between 3.75 and 1.0 ppm and between − 1.0 and − 4.0 ppm, where the CEST and rNOE pools, respectively, contribute to the spectrum.

**Figure 3:**
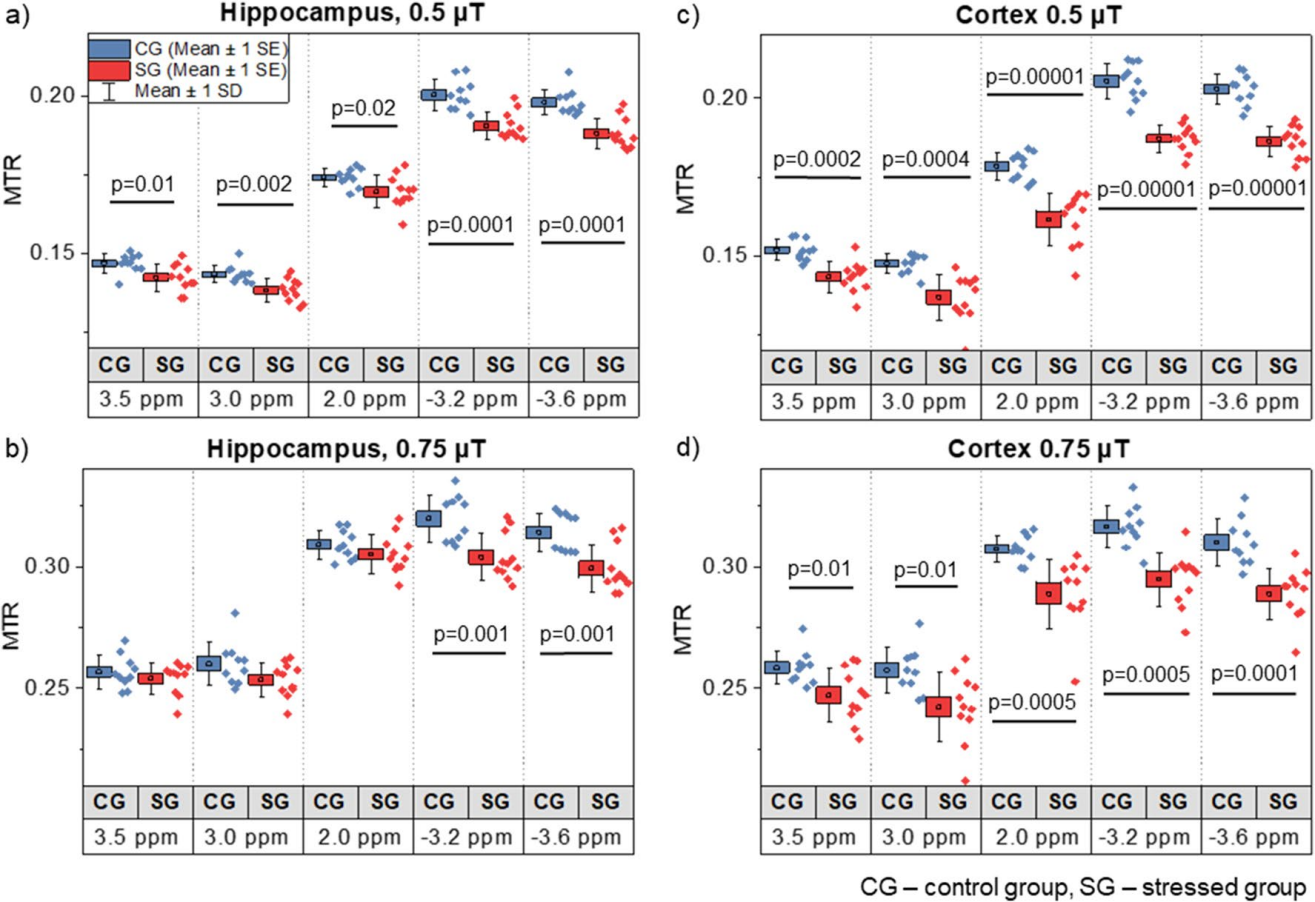
Comparison of magnetization transfer ratio (MTR) measured between groups in (**a**,**b**) hippocampus and (**c**,**d**) cortex with saturation B_1_s of 0.5 and 0.75 µT at frequency offsets of 3.5, 3.0, 2.0, − 3.2 and − 3.6 ppm. Statistically significant results are denoted by p-values derived from two-tailed Student’s t-test.

**Figure 4:**
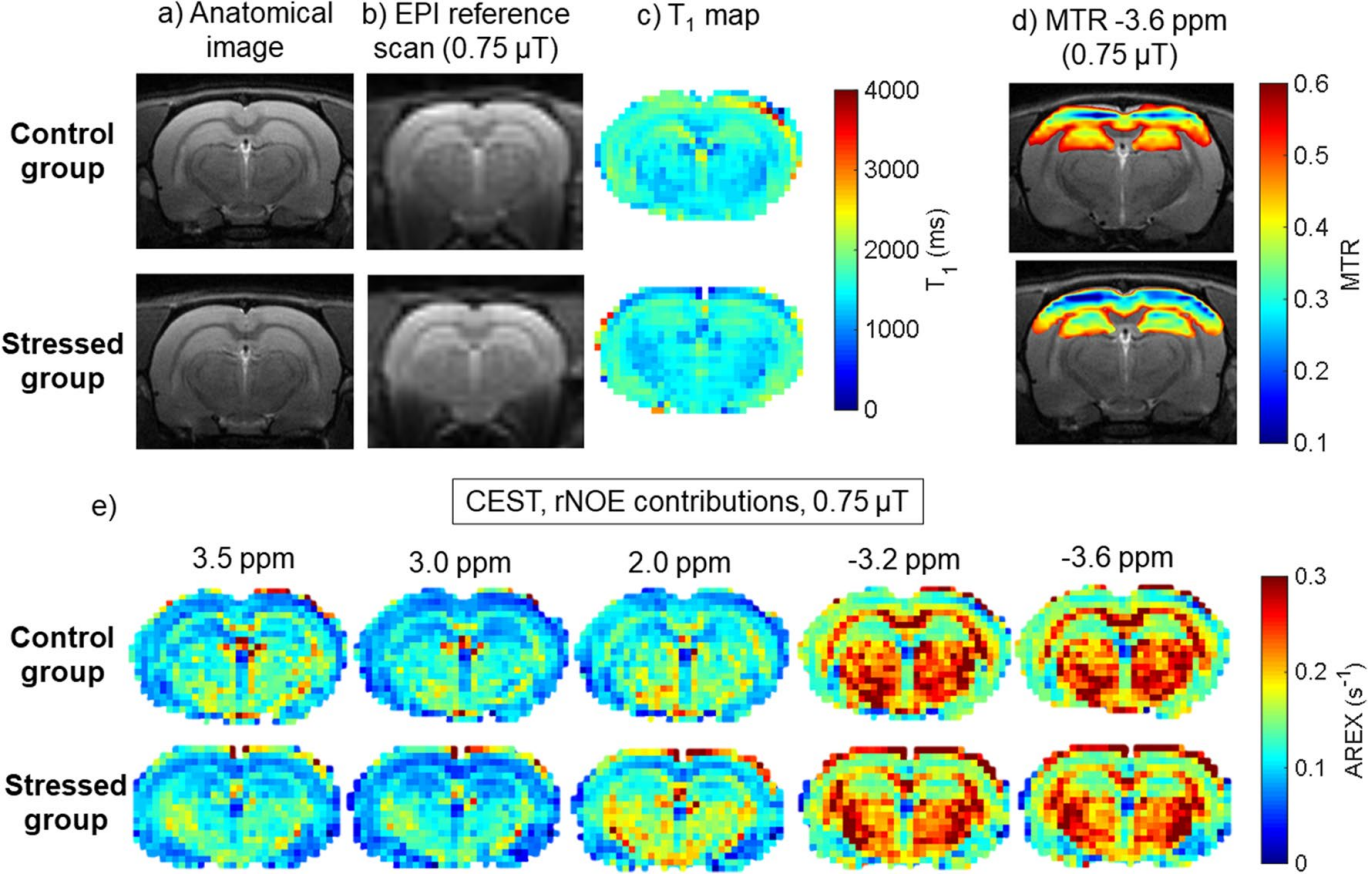
Images from the representative animals from control (first row) and stressed (second row) groups containing (**a**) anatomical T_2_-weighted RARE image, (**b**) EPI reference scan (B_1_ of 0.75 µT) (**c**) calculated T_1_ maps, (**d**) MTR maps with a saturation B_1_ of 0.75 µT at an aliphatic frequency offset (− 3.6 ppm) and (**e**) CEST and rNOE contribution maps covering whole brain area at all offsets of interest and B_1_ of 0.75 µT.

Parametric two-tailed t-Student test revealed that there was significantly lower MTR at all selected offsets in the hippocampus of the stressed vs. control groups with a B_1_ of 0.5 µT (Fig. 3a). Such differences occurred only at the relayed nuclear Overhauser effect (rNOE) offsets − 3.2 and − 3.6 ppm with a B_1_ of 0.75 µT (Fig. 3b). The magnetization transfer ratio in the region of the Z-spectra specific to the rNOE differed the most between groups with a maximum change of 1.5% with a B_1_ of 0.75 µT (p = 0.001). In the cerebral cortex, MTRs at all selected offsets, independent of B_1_, were significantly lower in the stressed group (Fig. 3c,d). The largest alterations were found at the rNOE offsets with a maximum change of 2.2% with a B_1_ of 0.75 µT at − 3.2 ppm (p = 0.0005). The differences between groups can be seen in the Fig. 4, where the MTR maps for the aliphatic offset of − 3.6 ppm (Fig. 4d) are superimposed into the anatomical images (Fig. 4a) and show decreases both in the hippocampus and cortex of the stressed group (the map calculated based on measurements with B_1_ = 0.75 μT saturation pulse applied to EPI sequence, from which exemplary reference scan is presented in Fig. 4b).

The observed longitudinal relaxation rate, R_1,obs_ (= 1/T_1,obs_), calculated from the T_1_ map (examples showed on Fig. 4c), and the two-pool MT model parameters are presented in Table 1. Parametric t-Student test revealed that R_1,obs_ was significantly faster in the stressed group, in both the hippocampus and cortex. None of the fitted parameters differed between the two groups of animals.

**Table 1:** The measured observed R_1_ *R*_1,obs_ (= 1/*T*_1,obs_) and estimated parameters of the two-pool MT model. Values (mean ± SEM) obtained from fitting the Z-spectra of control and stressed groups with saturation B_1_s of 3 and 5 µT in the hippocampus and cortex. CG denotes the control group and SG, the stressed group. To compare the results parametric two-tailed Student’s t-test or non-parametric Mann–Whitney U‐test (when data did not meet criteria of normal distribution) were performed. *p < 0.05.

Parameter	Hippocampus			Cortex		
	CG	SG	p-value	CG	SG	p-value
*R*_1,obs_ (1/s)	0.54 ± 0.003	0.56 ± 0.003	0.03*	0.52 ± 0.008	0.56 ± 0.005	0.02*
*T*_2,A_ (ms)	51 ± 1	52 ± 1	0.46	51 ± 1	50 ± 1	0.88
*R* (Hz)	50 ± 3	49 ± 2	0.83	47 ± 2	42 ± 3	0.29
*M*_0,B_ (%)	6.7 ± 0.2	6.7 ± 0.1	0.93	6.8 ± 0.2	7.8 ± 0.2	0.11
*T*_2,B_ (µs)	8.8 ± 0.1	8.7 ± 0.1	0.72	8.7 ± 0.1	9.0 ± 0.1	0.23

**Figure 5:**
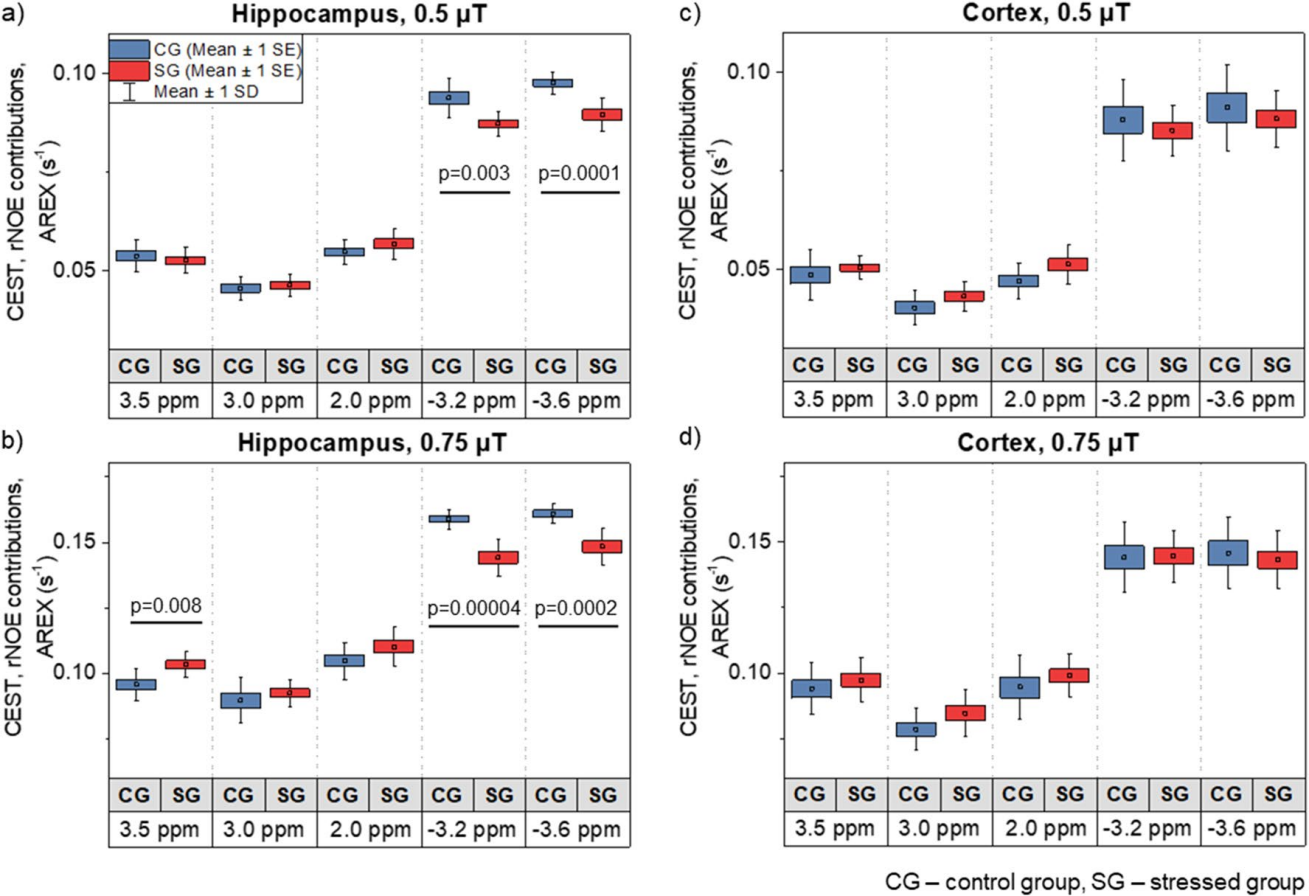
Comparison of the CEST and rNOE contributions after any T_1_ effect was eliminated between the control and stressed groups with saturation B_1_ of 0.5 µT and 0.75 µT in the (**a**,**b**) hippocampus and (**c**,**d**) cortex. In the hippocampus, stress-induced changes associated with the 3.5 ppm amide offset and − 3.2 and − 3.6 ppm aliphatic offsets were significant whereas, in the cortex, there were no significant differences between groups of animals in any of the chosen offsets. Statistically significant results are denoted by p-values derived from two-tailed Student’s t-test.

Figure 4e shows the maps and Fig. 5 the mean values of the CEST and rNOE contributions calculated using the AREX formula, which eliminates the T_1_, MT and direct water saturation effects from Z-spectra. In the hippocampus, the most pronounced changes between the control and stressed groups (revealed in parametric t-Student test) were found in the data within the rNOE range (examined at − 3.2 and − 3.6 ppm) with a B_1_ of 0.5 µT (Fig. 5a) and 0.75 µT (Fig. 5b). The largest decrease of 0.015 s^−1^ was found at − 3.2 ppm offset and B_1_ of 0.75 µT (p = 0.00004; Fig. 5b). There was also a significant increase in CEST amide (3.5 ppm) contribution from 0.096 to 0.103 s^−1^ (p = 0.008; Fig. 5b) after stress. In the cortex, the CEST contributions were slightly elevated in the stressed group at all examined offsets, with the opposite tendency from the rNOE contributions, similar to those in the hippocampus. However, none of the cortical changes were statistically significant (Fig. 5c,d).

**Figure 6:**
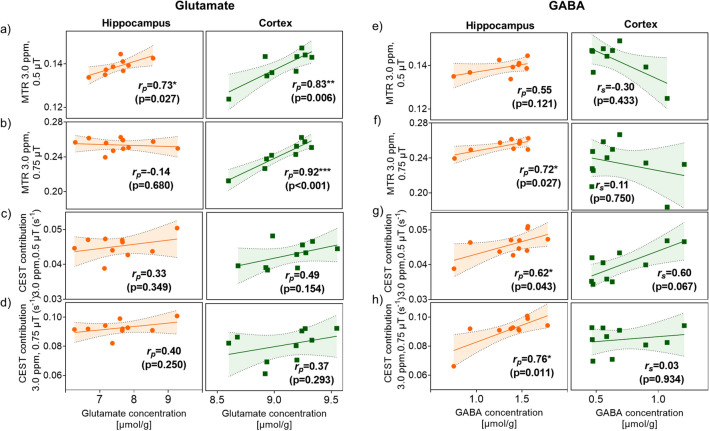
Correlation of (**a**–**d**) ELISA-derived glutamate and (**e**–**h**) GABA levels with MTR and CEST contribution measured at 3.0 ppm in the hippocampus (orange circles) and cortex (green squares) of the stressed group of animals. Shaded areas indicate the 95% confidence interval. The analysis was performed either with the parametric (Pearson’s, r_*p*_) or nonparametric (Spearman’s, r_*s*_) test.

Figure 6 shows scatterplots of MTR and CEST contribution at 3.0 ppm vs glutamate and GABA concentrations evaluated using a biochemical ELISA test, and Table 2 shows the parametric (Pearson’s, r_*p*_) and nonparametric (Spearman’s, r_*s*_) correlation coefficients. MTR measured with a B_1_ of 0.5 µT correlates with the glutamate concentration in the hippocampus (r_*p*_ = 0.73, p = 0.027; Fig. 6a) and cortex (r_*p*_ = 0.83, p = 0.006; Fig. 6a). In addition, the MTR measured with a B_1_ of 0.75 µT correlates strongly with glutamate concentration in the cortex (r_*p*_ = 0.93, p = 0.0001; Fig. 6b). CEST contributions with neither of these saturation B_1_s show no statistically significant correlations between the glutamate concentration in the hippocampus nor cortex (Fig. 6c,d) and ELISA. Turning now to GABA, hippocampal GABA concentration show significant positive correlation between MTR measured with a B_1_ of 0.75 µT (r_*p*_ = 0.72, p = 0.027; Fig. 6f) and CEST contribution at both B_1_s (B_1_ = 0.5 µT, r_*p*_ = 0.62, p = 0.043, Fig. 6g; B_1_ = 0.75 µT, r_*p*_ = 0.76, p = 0.011, Fig. 6h). While cortical GABA concentration had non-normal distribution, the Spearman’s nonparametric correlation analyses between GABA level and CEST metrics were taken into account, and none of them were found to be significant (Table 2).

**Table 2:** Parametric (Pearson’s *r*_*p*_) and nonparametric (Spearman’s *r*_*s*_) correlations analysis results. Correlation assessment performed between glutamate and GABA levels obtained in biochemical ELISA tests and MTR and CEST contribution calculated at 3.0 ppm in the cortex and hippocampus of the stressed group (*p < 0.05, **p < 0.01 and ***p < 0.001).

			Glutamate level	GABA level	
			Pearson's *r*_*p*_	Pearson's *r*_*p*_	Spearman's *r*_*s*_
Hippocampus	MTR: 3.0 ppm	0.5 µT	0.73*	0.55	
		0.75 µT	− 0.14	0.72*	
	CEST contribution: 3.0 ppm	0.5 µT	0.33	0.62*	
		0.75 µT	0.40	0.76*	
Cortex	MTR: 3.0 ppm	0.5 µT	0.83**		− 0.30
		0.75 µT	0.92***		0.11
	CEST contribution: 3.0 ppm	0.5 µT	0.49		0.60
		0.75 µT	0.37		0.03

**Figure 7:**
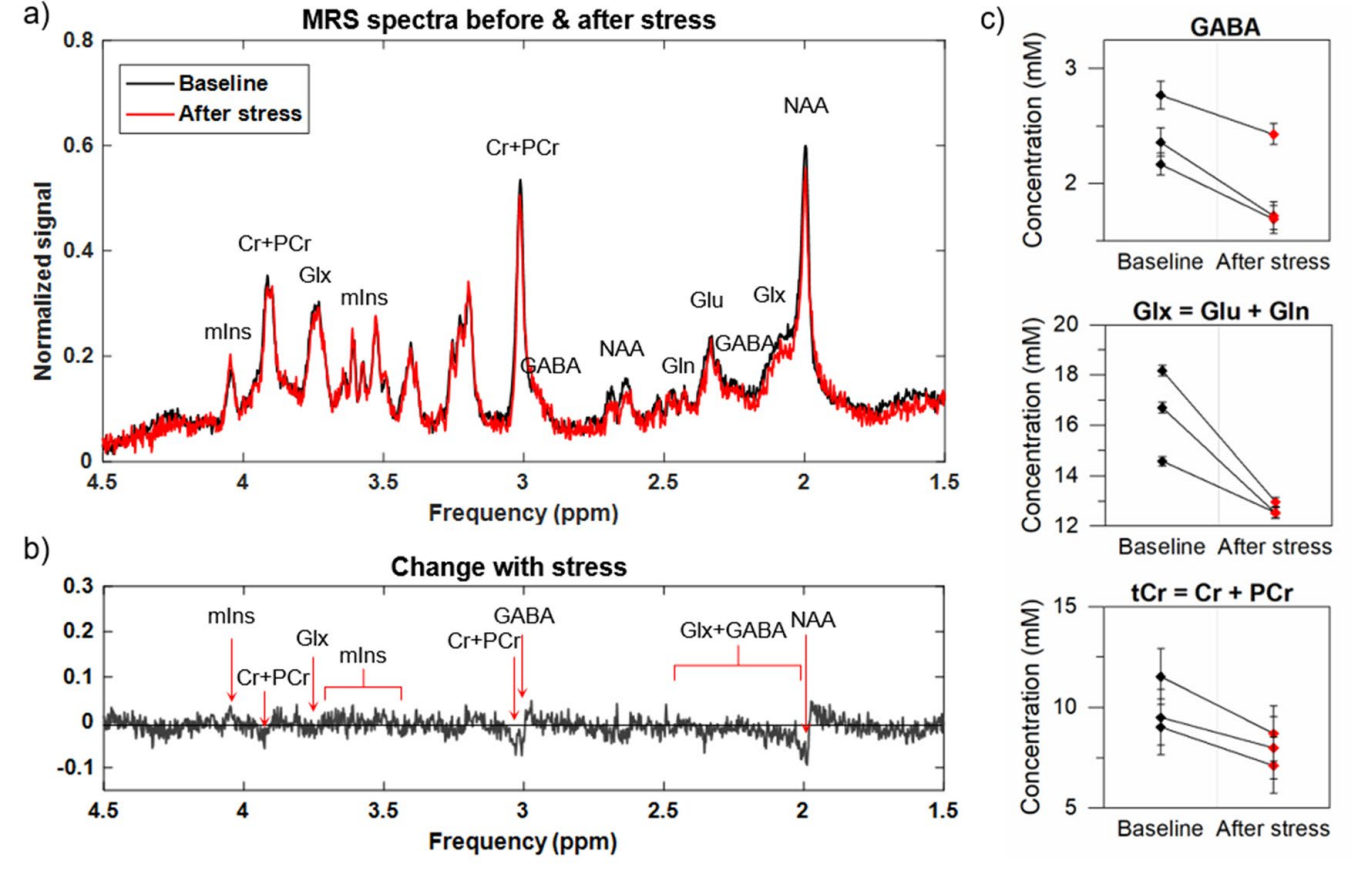
(**a**) Typical MRS spectra measured in a representative rat hippocampus before stress (black line) and after (red line). The lowering of the major peaks of *N*-acetyl aspartate (NAA) and total creatine (creatine + phosphocreatine, tCr = Cr + PCr), as well as of the signal within the 2–2.5 ppm range, where the Glx complex (glutamate + glutamine, Glx = Glu + Gln) and γ-aminobutyric acid (GABA) contribute to the spectrum are visible. The increase of myo-inositol (mIns) is also noticeable. (**b**) The residuals after subtracting the baseline from post-stress spectra. (**c**) The absolute concentrations of GABA, Glx and tCr were significantly lowered after stress, as revealed by t-test for paired samples (p = 0.03; 0.042; and 0.034, respectively). The error bars denote the fitting errors computed as SD of the model from the original data.

Figure 7 presents an illustrative MRS spectrum for an animal measured before and after stress (Fig. 7a) along with the difference between the baseline and post-stress measurements (Fig. 7b). Figure 7c shows changes in γ-aminobutyric acid (GABA), glutamine (Gln) + glutamate (Glu), together denoted as Glx, and total creatine (tCr) as the sum of creatine (Cr) and phosphocreatine (PCr), after stress. Finally, Table 3 includes all the metabolites with observed changes related to stress. The MRS measurements performed on three stressed animals revealed decreased signal in the range of total *N*-acetylaspartate (tNAA) containing *N*-acetylaspartate (NAA) and *N*-acetylaspartylglutamate (NAAG), Glx, tCr and GABA contributions to the spectrum after stress, as compared to baseline (Fig. 7a,b). The quantitative analysis confirmed significantly lowered concentration of these metabolites in t-test for paired samples (Fig. 7c and Table 3), while myo-inositol (mIns) was elevated after stress (Table 3).

**Table 3:** The MRS-derived absolute concentrations of metabolites (mean ± SEM), which significantly changes after stress. GABA, Glu, Gln, Glx, tCr, and total NAA (tNAA = NAA + NAAG) decreased, while mIns increased under stress. *p < 0.05.

Metabolite	Concentration at baseline (mM)	Concentration after stress (mM)	p-value
GABA	2.4 ± 0.2	1.9 ± 0.2*	0.03
Glu	11.0 ± 0.6	8.4 ± 0.2*	0.039
Gln	5.5 ± 0.4	4.3 ± 0.2*	0.049
mIns	6.9 ± 0.3	7.3 ± 0.2*	0.049
Glx = Glu + Gln	18.9 ± 1.1	14.6 ± 0.4*	0.042
tCr = Cr + PCr	10.0 ± 0.6	7.9 ± 0.4*	0.034
tNAA = NAA + NAAG	9.1 ± 0.4	7.4 ± 0.6*	0.048

## Discussion

There are several animal studies reporting changes in the brain neurochemical profile during depressive disorders using biochemical methods^13,38,39^ or MRS^15,40^. In this study, to evaluate brain damage caused by stress, we chose saturation transfer MRI metrics. MTRs, CEST and rNOE contributions to the Z-spectrum were analysed, for the first time, in relation to stress-induced alterations. This imaging modality was employed to study the presented model since most of the stress-affected brain metabolites are known to display the CEST effect^37^. We also performed MRS and biochemical ELISA test measurements to confirm our hypothesis that CEST, although less specific, is also sensitive to stress-induced metabolic changes, and that it may bring deeper insight into cerebral alterations in such conditions.

In this work, two groups of animals were examined: a control group and stressed group. The stressed group underwent an 8 week stress cycle with a protocol of chronic unpredictable mild stress (CUMS) to cause neuropsychological changes that corresponds to those of depressive disorders^7^. Animal behaviour was typical and not affected by any external factor before applying the stress protocol, showing exploration score per entry in the range of 0–12.2% in an elevated plus maze (EPM) behavioural test. CUMS was successful in inducing stress in the rats: only one individual from the stressed group demonstrated any interest in exploring area during the EPM test, while the remaining animals showed none (Fig. 1).

The MRI measurements clearly demonstrated that the brain metabolism of stressed rats was affected by CUMS. The most pronounced alterations were found within the rNOE range of the Z-spectrum (Fig. 2), which were evidenced by substantially lower magnetization transfer ratios MTRs (Fig. 3) upfield of the water resonance, but lower MTRs were also observed in a wide range of frequency offsets of the saturation pulse, in both the hippocampus and cortex of the animals in the stressed group. It should be noted, that MTR is the least specific metric used in this study, being influenced also by the T_1_/T_2_ ratio, macromolecular magnetization transfer (MT) and CEST effects. Thus, the observed alterations in MTRs had many sources^41^. We found that macromolecular pool characteristics estimated from fitting a two-pool MT model to the Z-spectra were not affected by stress (Table 1) indicating that MT does not change with stress, whereas the observed longitudinal relaxation rate R_1,obs_ was slightly faster in the hippocampus and cortex in the stressed group (Table 1) which might be indicative of decrease cell density or cellular loss^42^. Subsequently, the MT and direct water saturation effect contributions were removed from the Z-spectra allowing for evaluation of CEST and rNOE effect alone.

The modified AREX metric developed by Windschuh et al*.*^43^ for the evaluation of CEST and rNOE contributions was used in this study. The CEST results within upfield range of offsets complied with the MTR results (at − 3.2 and − 3.6 ppm offsets) in the hippocampus, but were less pronounced in the cortex. In the remaining analysed frequencies (at 3.5, 3.0 and 2.0 ppm), the trend was opposite. Although not significant, CEST contributions showed an increase of signal in the stress group as opposed to the MTR in corresponding offsets. The discrepancy of these results requires further investigation, thus it must be emphasized that CEST and rNOE contributions to the spectrum may contain signals from multiple overlapping chemical groups which could be one of the reasons.

The direct and MT effects were dominant contributors to the Z-spectra indicating that this metric was not particularly specific to brain metabolites. Removing these effects from the saturation transfer MRI data with the use of AREX method provided the parameter which could be potentially used for assessing subtle variations of brain metabolites’ levels, which accompany stress-induced depression. To the best of our knowledge, this the first time such specificity of a CEST-derived metric has been shown.

The analysis of correlation between MRI-derived parameters and additionally performed ELISAs of stressed group brains (hippocampus and cortex regions) together with supplementary MRS acquired from three stressed subjects (the SG_MRS_ group) shed light on the biological interpretation of the data. It should be emphasized that, in the stressed animals, we observed significantly lower MTR and Z-spectrum signal at 3.0 ppm, which is the offset corresponding to the amine groups of GABA, glutamate and glutamine^37,44^, which exhibited lower concentrations after stress in our MRS data (Fig. 7c and Table 3).

The MRS data showed post-stress decrease of glutamate and GABA, the two brain metabolites frequently reported as being affected by prolonged stress^13,15,38^. Note that both might contribute to the CEST spectra, albeit disentangling their contributions might be difficult because their CEST spectra might overlap. The decrease in GABA concentration has already been reported in the brain during depressive disorders^13,39^. However, phantom studies have demonstrated that GABA weakly influences the Z-spectrum at the saturation amplitudes used in this study^20,37^. GABA phantom studies have shown that the CEST peak of this metabolite is not clearly detectable with low saturation B_1_ amplitudes and is mostly pronounced at the higher B_1_ values (> 3 µT)^20^ which is mostly due to the faster exchange rate than found in other neurotransmitters^37^. Lee et al.^45^ have shown that, in a rat model of status epilepticus, the MTR asymmetry measured with B_1_ = 5.6 µT at 2.7 ppm in the hippocampus depends on GABA concentration changes induced by kainic acid, but authors also emphasize that the MTR asymmetry is a very non-specific measure cumulating effects from both sides of the z-spectrum. Nevertheless, our MRI data showed positive correlation with GABA levels assessed by ELISA in the hippocampus with CEST contributions measured at 3.0 ppm using both low B_1_s of 0.5 and 0.75 µT (Fig. 6g,h). However, this correlation might not be causative.

The literature is not consistent about the changes of glutamate levels in the brain in the rodent models of depressive disorders: some sources report a decrease^15,38^ while others show an increase with stress and depression^13,39^. Independent of this discrepancy, reported either in the cortex or hippocampus after prolonged stress, the neurobiological data consistently show that the glutamate stream via synaptic connections is disturbed in such condition^46,47^. Our previous study has showed that, in the CUMS rat model, the glutamate concentration in the hippocampal area is significantly lower^48^. In the present study, the change in signal (only MTR) measured with B_1_ saturation of 0.5 µT was less pronounced in the hippocampus as compared to cortex and not significant while using 0.75 µT saturation pulse at 3.0 ppm offset. Correlation analysis of the ELISA data showed, however, that the concentration of glutamate in the hippocampus and cortex of stressed group were significantly and strongly related to the results obtained from MTR analysis at 3.0 ppm (with both saturation B_1_ amplitudes in cortex and with 0.5 µT in hippocampus; Fig. 6a,b and Table 2). The same tendency was observed for CEST contribution assessed at 3.0 ppm, however the correlation was not significant (Fig. 6c,d).

On the other hand, it is worthwhile to mention the glutamate behaviour in saturation transfer experiments, in which it displays high exchange rates (5500 Hz) in phantoms at pH 7.0 and 37 °C and moderately high (2000 Hz) in the healthy rat brain^40^, which causes the Z-spectrum peak to coalesce with that of water (at 0 ppm) instead of appearing as a distinct peak at 3.0 ppm. This could be the reason why the CEST contribution extracted from Z-spectrum at 3.0 ppm did not correlate with biochemically assessed glutamate concentration. Rather, the contrast measured at 3.0 ppm may have significant contributions from the tails of the amide (3.5 ppm) and guanidinium (2.0 ppm) peaks^49^. The decrease in the MTR at 2.0 ppm (guanidinium) observed in the stressed group may correspond to lower concentration of creatine (Cr), which accounts for 66% of the Z-spectrum peak at 2.0 ppm in the rat brain^50^. It is to be expected, since decreased levels of total creatine tCr were found in our SG_MRS_ group (Fig. 7c and Table 2), and were already reported in the hippocampus and prefrontal cortex after chronic stress^38,40^.

It should be noted that CEST is less specific than MR spectroscopy, since the particular decrease of water signal pronounced in the Z-spectrum is a result of the exchange with protons originating from several compounds^41^. However, the sensitivity of CEST is much higher than other MRS methods, even 10^2^–10^5^-fold, depending on the molecule^41^, which makes the method usable even in subtle alterations caused by prolonged mild stress.

All the effects in the Z-spectra upfield from water (i.e., the rNOE signal at negative frequency offsets) were clearly related to stress-induced changes in the brain, as the reduction of the signal in hippocampus of the stressed group in all chosen measures was statistically significant within this range. One of the sources of the rNOE effect present in the Z-spectrum are lipids^51^, but while magnetization transfer, MT, which is mostly related to those structures did not vary with stress, we cannot clearly confirm what is the source of the substantial loss of the rNOE signal from brain after CUMS protocol. In hippocampus, progressive deterioration of tissue is induced by prolonged stress^47^. In such circumstances, increased glycine and glutamate concentrations induce intensified activation of the *N*-methyl-d-aspartate (NMDA) receptor, one of the glutamate receptors, densely located in hippocampus, cortex, striatum and limbic systems^46^. Such conditions of overactivity in the long run (e.g., during chronic stress) lead to cytoskeletal degradation by the intensified calcium stream, protein misfolding and oxygen radical generation, which collectively lead to neuron death and hippocampal shrinkage^46,47^. As the NMDA receptor is also densely distributed within the cortex^46^, such changes may also apply to this region. Although we do not have enough biological data to confirm this, it is plausible that some of these processes contribute to lowering of rNOE metrics after prolonged stress observed in this study in hippocampus and cortex. This conclusion may be supported by the increase in the observed longitudinal relaxation rate, R_1,obs_, found in both the hippocampus and cortex of the brains of stressed animals (Table 1). Such changes are usually observed in the process of brain ageing due to progressive decrease of cerebral water content^52^. The increased R_1_ found in the brains of stressed rats was consistent with other studies, where the similar trends are observed in hippocampus of depressed patients and the change is even more pronounced in elderly brain^53^. Intensified water volume regulatory processes are also present during inflammation, which was reported to be a result of chronic stress in the brain^15^. This might be the case in our study, where MRS data showed increased hippocampal concentration of mIns, a metabolite responsible for osmoregulation^15,54^.

Further development of the CEST protocol would be necessary for transferring the methodology to human studies. It is worth mentioning that a clinical CEST protocol may contain only one or a few offsets of interest which will result in a substantial reduction of study time. This quick protocol could be repeated multiple times in different stages of disease or treatment process to monitor even subtle changes in brain metabolism that occur during depressive disorders or different dysfunctions. The information obtained from that kind of study would not be as specific as from MRS, but the scan would be shorter and more sensitive to brain metabolism alterations. Also, to meet the restrictions in specific absorption rate in patients, the use of pulsed CEST should be considered^55^, instead of the continuous wave radiofrequency irradiation used in this study.

## Methods

### Animal model preparation and behavioural testing

The experiments were performed at the Experimental Medicine Center at the Medical University of Lublin, Poland, and reported in accordance with ARRIVE guidelines. The experimental animal protocol was approved by the Local Ethics Committee for Animal Experiments of the University of Life Sciences in Lublin, Poland. All the procedures were compliant with the Guide for the Care and Use of Laboratory Animals of the National Research Council (8th edition, 2011)^56^. Twenty four male Wistar rats (200–240 g) were used in the study. The animals were kept in polypropylene cages at 22 ± 1 °C with 50 ± 5% relative humidity, a 12 h light–dark cycle and free access to food and water. After 2 weeks of acclimatization and handling, the animals were divided randomly into two groups: control (n = 10) and stressed (n = 14). Animals from control group were double-housed and animals from stressed group were single-housed to avoid aggressive behaviour between rats kept together as a consequence of prolonged stress.

The stressed group underwent the chronic unpredictable mild stress (CUMS) protocol, a standard animal model of depressive disorders^57,58^. For 8 weeks, the animals were exposed daily to one of seven different stress factors: 24 h water deprivation; 24 h food deprivation; 5 min cold swimming (at 4 °C); overnight illumination; 4 h of 45° cage tilt; 24 h in a wet cage and ~ 50 min in a cold environment (at 4 °C).

Before the onset of the stress protocol, all animals were subjected to a behavioural test to determine baseline performance. The elevated plus maze (EPM), first described by Pellow and File^59–61^, was selected as it has been demonstrated to be affected by the CUMS protocol^62^, does not resemble any of the stressors used, and unlike common tests for depression such as a forced swim test^63^ or Morris water maze^64^, EPM does not subject animals to undue stress which could affect the CEST-MRI results in control animals. EPM consists of placing a rat in a cross-shaped maze with two walled-in (dark) and two no-wall (lighted) arms and raised above the ground. The animals were placed initially in the dark space and left in the labyrinth for 5 min. The device was constructed so that the rodent could move freely between dark and light regions. The number of entries into the light areas, and the time spent within them was measured. As a quantitative measure of stress, the percentage of time that an animal spent exploring the environment (behavioural score) per entry was assessed. The behavioural test was repeated again for the stress group after the end of the stressing period, one day before MRI.

### MRI and CEST

All animals were scanned after eight weeks of the stress protocol at 7 T MRI (70/16 Pharma Scan running ParaVision 6.0.1, Bruker BioSpin, Ettlingen, Germany) using a 72-mm inner diameter volume coil (T20117V3) for transmit and a 20-mm surface loop coil (T116344) for receive. For the CEST experiments, twenty one animals were used, divided randomly into two groups: control (n = 10) and stressed (n = 11). See below for the remaining three stressed animals. The entire experiment lasted approximately 2.5 h per animal from the induction of anaesthesia until the end of imaging. Anatomical images were acquired in the axial plane using 2D T_2_-weighted rapid acquisition with refocused echoes sequence (RARE, TR/TE_eff_ = 2500/33 ms, FOV = 30 mm × 30 mm, slice thickness = 1 mm, matrix = 256 × 256, RARE factor = 8, bandwidth = 52 kHz, averages = 2, time = 2 min). The slice of interest for the single-slice Z-spectrum measurement was selected with the reference to the Scalable Brain Atlas^65^. The images covered an axial section of the ventral part of the hippocampus and cerebral cortex located directly above the hippocampus (Fig. 4a). The alignment of this section is 0.61 mm anterior to the anterior commissure according to the CBWJ13 MR-histology rat atlas at age P80 (slice 65 ± 0.15 mm)^66^. Map Shim was performed for B_0_ correction in an ellipsoidal volume covering the whole brain in the chosen slice.

Z-spectra sensitive to CEST, rNOE and magnetization transfer (MT) contributions were acquired from a single axial slice using a saturation transfer-prepared EPI sequence (Fig. 4b) with block saturation B_1_ pulses of 0.5 and 0.75 µT peak amplitude (139 offsets each). Reference scans were performed with saturation at 200 kHz offset (667 ppm) after every five images for baseline correction. Measurements were performed at linearly spaced frequency offsets between 6 and − 6 ppm. In addition, two more Z-spectra, which are sensitive mostly to MT, were measured with B_1_ peak amplitudes of 3.0 and 5.0 µT (at 32 logarithmically spaced offsets between 300 to − 300 ppm each). In addition, a water saturation shift referencing (WASSR)^67^ Z-spectrum, which is sensitive only to the direct water saturation effect (DE), was acquired with a B_1_ peak amplitude of 0.1 µT at 24 linearly spaced offsets between 0.5 and − 0.5 ppm. For all Z-spectra, the duration of the saturation pulse was 4900 ms and remaining parameters were as follows: TR/TE = 5000/37 ms, FOV = 30 mm × 30 mm, slice thickness = 1 mm, matrix = 64 × 64, bandwidth = 20 kHz, averages = 2. 3D FLASH with high flip angle (TR/TE = 200/3.5 ms, FOV = 30 mm × 30 mm, slice thickness = 1 mm, matrix = 64 × 64, bandwidth = 50 kHz, averages = 1, time = 2 min and 20 s each, flip angles range = 130°–220°) was performed for B_1_ correction. Finally, longitudinal relaxation rate, R_1_ (1/T_1_) maps, which were further used for the quantitative MT model fitting, were calculated from five inversion recovery RARE scans (TR/TE_eff_ = 10,000/6 ms, TI = 30, 230, 650, 800 and 5000 ms, FOV = 30 mm × 30 mm, slice thickness = 1 mm, matrix = 64 × 64, bandwidth = 67 kHz, averages = 1, time = 2 min each).

### Supplementary MRS

Because of the long acquisition time of the CEST protocol, the remaining three animals from stressed group were used for a separate MRS measurement, denoted SG_MRS_, to verify metabolic profile changes after stress. MRS was performed twice: before stress as a baseline and after the CUMS protocol.

Prior to voxel positioning for MRS, three-plane T_2_-weighted RARE scans were acquired: TR/TE_eff_ = 2500/33 ms, RARE factor = 8, matrix size = 256 × 256, slice thickness = 1 mm. The 2.0 × 2.0 × 5.5 mm^3^ volume of interest (VOI) was placed over the right hippocampus. The B_0_ magnetic field inhomogeneity was corrected using shim adjustments from ParaVision’s LocalisedShim procedure. The achieved full width at half maximum (FWHM) of the waterline was between 8 and 9 Hz.

MRS spectra were acquired with the point resolved spectroscopy (PRESS)^68^ sequence: TE = 16.66 ms (TE_1_/TE_2_ = 8.87/7.79 ms), TR = 2500 ms, spectral bandwidth = 3 kHz, 4096 data points were collected, and 1024 averages used. The water signal was suppressed using seven CHESS schemes of combined variable power RF pulses with optimized relaxation delays (VAPOR)^69^. VAPOR pulse amplitudes were adjusted manually for each animal to achieve optimal water suppression. Additional non-water-suppressed spectra were acquired for an absolute quantitation of metabolites concentrations in further analysis.

### Animal monitoring

In order to minimize the effects of food intake on the neurotransmitters levels, the animals were deprived of food for about 6 h before scanning. The animals were anesthetized with 3.5% isoflurane in oxygen flowing at 0.7 L/min for induction and 1.7–2.2% for maintenance. The anaesthetic concentration was adjusted to keep the respiratory rate at ~ 50 bpm. The respiratory rate was monitored using a pillow placed under the belly (Small Animal Instruments, Inc., Stony Brook, NY, USA). Body temperature was maintained at 37 ± 0.2 °C using a circulating, heated water bed system and endorectal temperature probe (Small Animal Instruments, Inc.).

### ELISA

After MRI, while maintaining anaesthesia, animals from the stressed group were sacrificed and the brains were sectioned into the hippocampus and cortex. Collected brain tissues were stored at − 80 °C until analysis. Brain tissue was ground with a handheld homogenizer VDI 12 (VWR International, Gdańsk, Poland). Extraction of metabolites was carried out using perchloric acid and sodium hydroxide^70^. Levels of glutamate and GABA were assessed using enzyme-linked immunosorbent assay (ELISA) research kit (LDN, Nordhorn, Germany)^70^.

### Image preprocessing and model fitting

**Figure 8:**
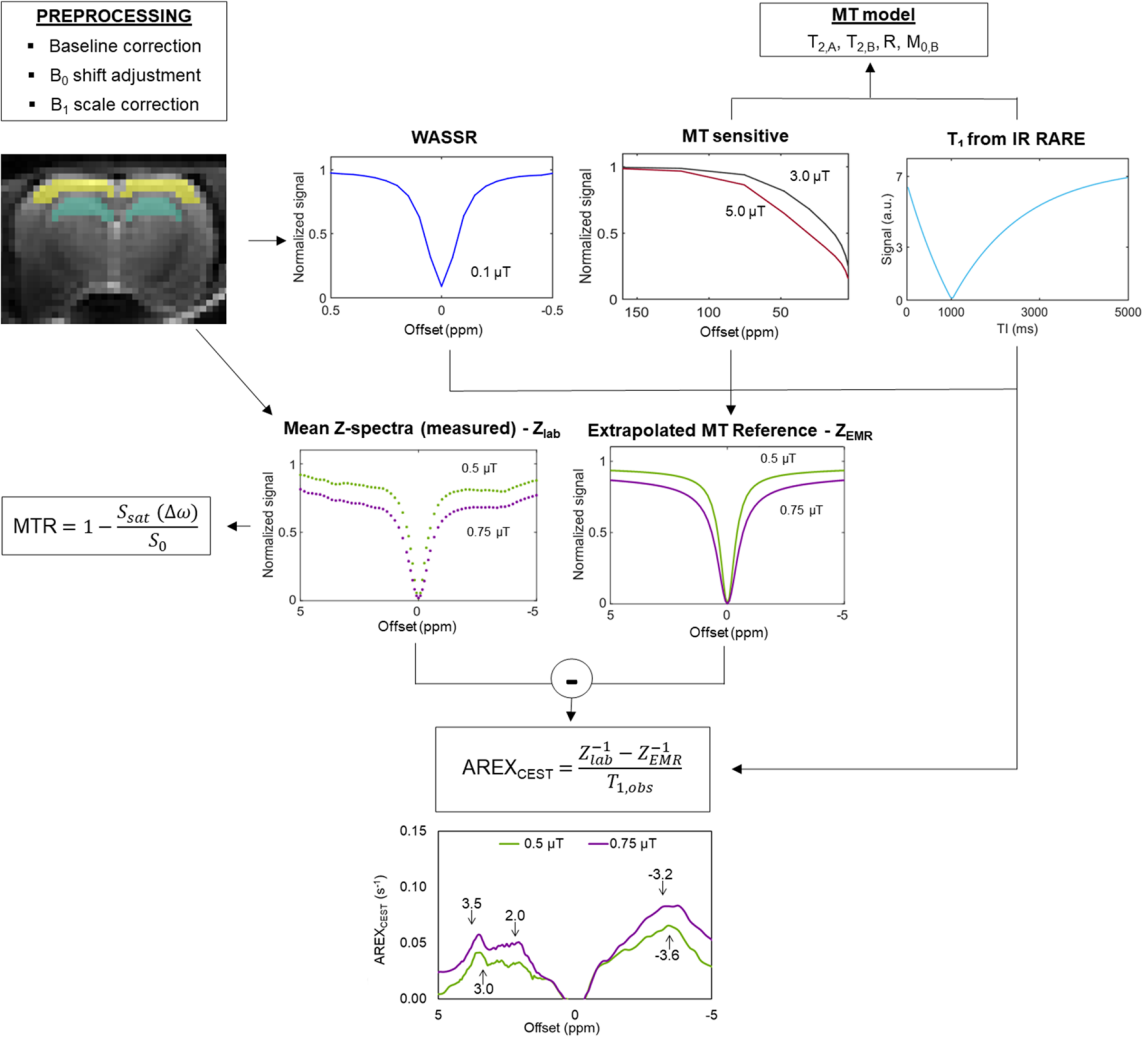
Graphical illustration of the analysis pipeline. First, the preprocessing steps including baseline correction, B_0_ shift adjustment and B_1_ scale correction were made. From two regions of interest (cortex and hippocampus), five mean Z-spectra with B_1_ amplitudes of 0.1, 0.5, 0.75, 3.0 and 5.0 µT were generated. The magnetization transfer ratio (MTR) was calculated from Z-spectra acquired with B_1_s of 0.5 and 0.75 µT (*S*_sat_, *S*_0_ are the signals measured with and without saturation pulse, respectively). The T_1_ map calculated from inversion recovery RARE images was used with MT sensitive Z-spectra (B_1_s of 3.0 and 5.0 µT) and DE-sensitive WASSR spectra (B_1_ of 0.1 µT) for two-pool MT model fitting. The estimated MT model parameters were used to calculate extrapolated MT reference spectra (*Z*_EMR_) for B_1_s of 0.5 and 0.75 µT, representing the semi-solid-originated MT effect. Measured Z-spectra (*Z*_lab_) (with the same B_1_s of 0.5 and 0.75 µT) were subtracted from extrapolated MT reference spectra (*Z*_EMR_) providing CEST and rNOE contributions. The apparent exchange-dependent relaxation (AREX_CEST_) equation was used to eliminate T_1_ effect from CEST and rNOE contributions. Finally, MTR, CEST and rNOE contributions were calculated for five selected offsets (3.5, 3.0, 2.0, − 3.2 and − 3.6 ppm).

Figure 8 presents the preprocessing steps and the pipeline of extracting MTRs and CEST and rNOE contributions for five selected frequency offsets (3.5, 3.0, 2.0, − 3.2 and − 3.6 ppm) using a two-pool MT model and AREX_CEST_ equation.

For CEST data analysis, two anatomical areas were selected: the hippocampus and cerebral cortex (Fig. 3). Regions of interest were drawn manually using FSLView (version 3.2, University of Oxford) and, from those areas, the mean Z-spectra at saturation B_1_ amplitudes of 0.1, 0.5, 0.75, 3.0 and 5.0 µT were generated.

MRI data were analysed in a manner similar to our previous study^48^ using MATLAB (version R2016b, The MathWorks, Natick, MA, USA). Briefly, the reference scans were used to normalize the signal and correct the baseline drift in all Z-spectra. B_0_ correction was performed spectrum-wise as follows. The sum of two Lorentzians (representing the MT and DE pools) was fitted to each Z-spectra with saturation B_1_s of 0.5 and 0.75 µT in the range of [− 1, 1] ppm to re-centre Z-spectra such that the water saturation peak was at 0 ppm^71^. Similarly, one Lorentzian (representing the DE pool) was fitted to the Z-spectrum with a saturation B_1_s of 0.1 µT and the Z-spectrum re-centred. A T_1_ (= 1/R_1_) map was calculated from the inversion recovery RARE images^72^.

For semi-quantitative evaluation of the data the magnetization transfer ratio (MTR) was evaluated, defined as:

1 $${\text{MTR}}= 1- \frac{{S}_{\text{sat}}}{{S}_{0}},$$

where *S*_sat_ and *S*_0_ denote signal measured with and without saturation pulse, respectively. Here, we reported MTR for five offsets, corresponding to peaks in the Z-spectra: 3.5 (amide CEST), 3.0 (amine CEST-the contribution to CEST contrast at this offset may not be due to amine; see the Discussion for more details), 2.0 (guanidinium CEST), -3.2 and -3.6 ppm (both aliphatic rNOE).

Since MTR depends on the magnetization transfer and direct effects, further analysis was also performed to isolate CEST and rNOE contributions. To do so, first, the Z-spectra with saturations B_1_s of 0.1, 3, and 5 µT and the R_1_ map were fitted using a nonlinear least-squares solver (running the trust-region-reflective algorithm) to a two-pool MT model^73^, which described the lineshape of the semisolid macromolecular pool using a super-Lorentzian and the free water pool using a Lorentzian. Four quantitative MT parameters were evaluated: the transverse relaxation times of the free liquid (*T*_2,A_) and macromolecular (*T*_2,B_) pools, initial magnetization of the macromolecular pool (*M*_0,B_), and magnetization exchange rate from the macromolecular to water pool (*R*). MT-only Z-spectra (Z_*EMR*_) were extrapolated using these estimated model parameters to the B_1_s of 0.5 and 0.75 µT and frequency offset matching those of the measured Z-spectra (Z_*lab*_)^71,74^. The apparent exchange-dependent relaxation (AREX_CEST_) calculated by the following formula was used to eliminate any MT exchange, direct water saturation, and T_1_ effects to yield only the CEST and rNOE contributions (Fig. 3)^75–77^:

2 $${\text{AREX}}_{\text{CEST}}=\frac{{Z}_{\text{lab}}^{-1}-{Z}_{\text{EMR}}^{-1}}{{T}_{1\text{,obs}}},$$

where *Z*_lab_ is the measured Z-spectrum (with B_1_s of 0.5 and 0.75 µT), *Z*_EMR_ is the extrapolated MT reference and *T*_1,obs_ is the measured T_1_. Finally, MTR, the quantitative MT parameters, R_1_ relaxation rates, CEST and rNOE contributions and mean Z-spectra were compared between control and stressed groups.

For the analysis of MRS data form SG_MRS_ group, the software jMRUI was employed (version 6.0, MRUI Consortium, http://www.jmrui.eu) with analysis in the time domain^78^. A basis set of 22 metabolites was simulated with the NMRScopeB plugin (version 2.1^79^) using the PRESS sequence parameters and magnet characteristics as input and then fitted to the signal. The fitting errors for each metabolite were computed by jMRUI as the standard deviation, SD of the model from the original data and was expressed as a percentage. jMRUI also provided an evaluation of the SNR as the ratio of the maximum in the model spectrum to twice the residuals. The unsuppressed water MRS signal was used to normalize the fitted signals of metabolites to water content and to calculate absolute concentrations of metabolites in tissue (expressed in millimolar).

### Statistical analysis

All statistical analyses were performed in Statistica 13 (StatSoft Inc., Tulsa, OK, USA). EPM behavioural test results in the SG group were compared using non-parametric Wilcoxon test for paired samples. The two-tailed Student’s t-test for independent variables was used to compare parameters between the CG and SG groups. If the distribution of results did not meet the assumptions of a normal distribution, a nonparametric Mann–Whitney U‐test was performed. The before and after CUMS concentrations of metabolites measured by MRS in SG_MRS_ group were compared with the use of t-test for repeated measures (for paired samples). The 3.0 ppm MTR and CEST contribution dependency on ELISA-derived concentrations of metabolites (GABA and glutamate) was assessed with Pearson’s correlation or non-parametric Spearman’s monotonicity analysis when data exhibited non-normal distribution. When rejecting outliers, Chauvenet's criterion was applied. The results were considered significant at p < 0.05.

## Data Availability

The data that support the findings of this study are available from the corresponding author upon reasonable request.
